# Enhance Software-Defined Network Security with IoT for Strengthen the Encryption of Information Access Control

**DOI:** 10.1155/2022/4437507

**Published:** 2022-10-03

**Authors:** Vrince Vimal, R. Muruganantham, R. Prabha, A. N. Arularasan, P. Nandal, K. Chanthirasekaran, Gopi Reddy Ranabothu

**Affiliations:** ^1^Department of Computer Science & Engineering, Graphic Era Hill University, Dehradun, Uttarakhand, India; ^2^Department of Information Technology, TKR College of Engineering & Technology, Meerpet, Telangana, India; ^3^Department of ECE, Sri Sai Ram Institute of Technology, Tambaram West, Chennai, Tamil Nadu 600044, India; ^4^Department of Artificial Intelligence and Data Science, Panimalar Engineering College, Chennai, India; ^5^Department of Computer Science and Engineering, Maharaja Surajamal Institute of Technology, New Delhi, India; ^6^Department of ECE, Saveetha School of Engineering, Saveetha Institute of Medical and Technical Sciences, Chennai, Tamil Nadu, India; ^7^Wachemo University, Hosanna, Ethiopia

## Abstract

The Internet of Things (IoT) is legitimately growing quicker. The operators have already started setting up a diligent infrastructure for these gadgets. Various technologies need to be developed for this type of sensor, including enterprise safety initiatives. This paper covers the stability routing protocol, which assumes an assessment of credibility in gadgets and packet flow. To build reliable Software-Defined Network (SDN) routes, build on the trust between network element flows and Quality of Service (QoS) or energy conditions. The SDN architecture is used for the Cognitive Protocol Network (CPN) technical platform to increase the energy level. Stochastic Neural Networks (SNNs) are accredited with information extracted from perceptual packets and make decentralized decisions. The proposed network infrastructure is designed and integrated into the SerIoT techniques to strengthen IoT encryption for information access control. The versatility of the technology is to circumvent the unpredictable connectivity of the system and the node decreases in terms of potential cryptographic capacity, limited interval, a target node, and deterministic energy. Based on factual statistical data, appropriate marketing generates an end-to-end antitheft solution that meets a set of predetermined circuit restrictions. A study must collaborate by demonstrating numerous flaws due to the obvious instability of clusters, which is essential for the efficiency of the platform.

## 1. Introduction

Stability in IoT is no longer perceived as a secondary concern, but rather as a relevant concept throughout the development of technological infrastructure or software applications. Intimate location, biometric data, or business information are among the most dangerous data acquired by electronic objects. Interconnections extract data that would be used to control urban and industrial infrastructure [1]. The deteriorating IoT sector raises concerns about the reliability of the IoT devices or the connections that send information. If verified or corrected, then aggressions have an enormous cost to the distribution system, including the lack of credibility in a competitive world [2]. Manage devices that have hacked abandonment risk based on customer or end-user credibility to increase overhead, legal costs, increased electricity usage, operating costs, and CO2 damage [3].

Partitioning is one of the most important steps to ensure secure Internet access. As a result, hackers focus on targeting 14,000 attacks such as drains in data loss, income loss, and reputational damage in 2017. Navigation assaults include a malware on the delivery service, intrusion on the morphological provider, assaults on several path services, and facilitation of authenticity. It was categorized in [4, 5] and intends to exhaust network capacity and espionage. Track protection requires considerable investigation. Configuration as a technique of assault prevention gets very little attention in the search for the difficulty that arises in ad hoc networks. A new website on protection-sensitive sequencing in SDN base stations [2] is already disclosed.

Traffic Analysis and Fault Detection (TA/FD) are surveillance or mitigation strategies commonly used in computer systems. It stipulates the adequate encryption methodology used to protect the most vulnerable components of the network where TA/FD is not able to make a definitive remediation option [6]. Leads potentially vulnerable parts to the transportation of dangerous goods system to provide recreation for thorough investigation or redirect flows [7].

Rerouting the importance of navigation in preventing assaults in computer systems led us to include the administration of security circuits. As a key component of SerIoT (Reliable or Protect Wearable computing) [8]. Collaborates on unique channel management approaches for SDN networks that focus primarily on the monitoring and disclosure of online perceptual security [9].

Accumulate numerous safety statistics or have clues about restricted insurance of specific vehicles or hubs that redirect pedestrians presumed to be components of a cyber incident to a less immediate alert [10]. Adapting protective trails or omitting the server are all examples of trail monitoring based on stability. To achieve improved durability assaults as a response to newly established techniques or methodologies, raising a measure of assurance for either network provider or network consumer gadgets [11]. One reason for focusing on SDN's substantial progress in investigation or installation, penetration testers are still in their infancy for comparatively small and talented innovation.

More specifically, the EU's FP7 NEMESYS research allowed researchers to study the stability of mobile operators. It includes the centralized controller component of the functioning of the mobile network, and several attacks specifically target it [12, 13]. Vulnerabilities in infrastructure technologies that exploit the research when it is functioning correctly or in danger. Messaging services used to leverage data, information, and instructions from various parts of the network are all presumed in the further investigation of surveillance of malware processes [14].

In [15], a relevant current study on cybercrime in Europe is reviewed on numerous initiatives funded by the European Commission. Cybercrime for mobile communications is a serious barrier to all aspects of information systems [16]. While the majority of modern mobile devices offer WiFi access from other cellular routers, the emerging security controls should be continuously monitored on the network and dominate aircraft levels in smartphones. Consequently, a previous survey [17] examined the use of Artificial Neural Networks (ANNs) and computer vision technologies to address this concern. Research [18] focuses on assaults on the aircraft triggering the foundation network, which has a direct impact on the mobile service provider or the end consumer. The NEMESYS initiative has dispelled many of these difficulties by leveraging the methodologies of queuing theory.

Concentrates [19] on the integrity of communications and information delivery for European regional or national medical systems which are equipped. European explorers occasionally need to seek medical coverage in another European country, health computing platforms should be able to get enough virtual patient records. Stability in the residential IoT industry is addressed in [20], which focuses on the architecture of a robust residential central server. It includes assault identification techniques and evaluation of assault tactics that attempt to deplete energy production from products by exhausting their devices. The SerIoT initiative began in January 2018, and further information may be found [21]. To persuade or investigate attacks, it should develop and execute phishing emails on virtual servers.

## 2. Materials and Methods

SernCPN, a CPN-based network that affects custom dispersed IoT systems, would be discovered through research. This would use calibration to create system self-awareness by implementing the SDN built on the CPN. This SDN would use Cognitive Packet (CP) to find secure multihop paths with a reliable Internet connection. And, evaluate their security and reliability, supervised learning with SNN to improve the overall performance of the system. It would include the three objectives of safety measures, good service, and lower energy consumption. Multiple sets of SernCPN networks can be credited together using a final integration node and adaptable links to Grid or Cloud platforms for network data management and analytics.

In addition, AI and IoT could be leveraged in coordination to remove the ZeroDay assault. A zero-day exploit exposes an organization to previously unexplored vulnerabilities, and there is no way to build a counterforce. A zero-day exploit exposes an organization to previously uncharted vulnerabilities, and there is no way to build a counterforce. Artificial intelligence (AI) would be designed to monitor the rate of potential malware with features derived from relevant services that predict the probability of traps. Meanwhile, AI and IoT could be used synergistically to impede Advanced Persistent Threats (APTs) based on adaptation classification methods [22]. In one study [23], one of the cases involving the integration of IoT and AI for enhanced security is explored. The authors of this research guideline an AI approach to reduce banking crime in an IoT scenario. This article highlights how IoT leverages recruitment and evolution algorithms to determine customer behavior in securities fraud prevention, and credit card theft, using an approach like BOAT [24]. According to Choi and Lee's research, using an ANN could accurately detect a forgery in actual time.

## 3. Design Theory of Secure IOT System

An IoT system's creative process is especially important in industrial platforms since it dictates which equipment a company would navigate to cognitive computing [25]. These gadgets, in turn, are part of the enormous particles of data surveillance of the universe. The same reliability of enterprise-critical data would be dictated by the installation of IoT systems. IoT's creative process would have implications for safety protocols because it depends on a combination of offspring. Include a well-articulated communications plan, reinvent accountabilities when developing a security protocol plan, and keep them informed of key innovations to ensure information security [26]. In addition, Protected IoT's research approach is based on three primary criteria. The first step involves connecting direct input devices or embedded annotation devices to the main cloud server. This includes multi-factor authentication of responders and verifying equipment attempting to enter the facility. A second step would allow retrieving, optimizing, and delivering substantially the data of different instruments in the gadgets. This is achieved by choosing the optimal data or model that is acceptable to the cloud-based industrial IoT architecture.

One of the goals of the research is to build and test a reliable SDN-related IoT fiber network and an intelligent controller with Internet perceptual monitoring and disclosure. The ability to build and revise routes in an adaptive way to increase security for IoT devices and business customers while preserving the near-optimal quality of goods. CPN methodology introduced in [27], is used for online perceptual supervision and route surveillance. Numerous studies, discuss the principal components or functionality of the CPN router. The SernIoT “SernIoT CPN network” or SernCPN smart SDN network starts with some of the concepts described in [28].

The study of a revolutionary approach to transmission involves related concepts. Although the term may seem ambiguous in practice, many researchers have already defined confidence in a form that is also adopted for the study. A “sustainability intensity” of nodes within a network is considered to be trusted [20]. According to this description, credibility is defined as the likelihood that collaborating nodes in a network will adhere to the network's regulatory frameworks. It would not violate the security criteria of anonymity, stability, access, legitimacy, and nonrepudiation.

SernCPN would be a secure multipurpose network platform, concerned about the QoS or energy that could be used in various scenarios, including the IoT domain [20].

1. A digital IoT platform would be separated from the contractor's base network
2. Overlay on the Internet, in which regular Internet capabilities are used instead of wired connections
3. Local communication through a major IoT network

SernCPN takes the baseline SDN method and enhances it. It features data and configuration control plans and interfaces between them using the OpenFlow protocol. Priority-based eligibility guidelines should be used to make decisions about data flow:

1. Security & Stability: information must be provided securely, with the least impact of being blocked and lost (due to deliberate or accidental defeat). This includes safeguards against hacking the switch and regulator and measures to pass erroneous data to a central device (or other networking devices).
2. QoS: manifestations such as flow, latency, and fluctuation are important criteria in determining packet arrival routes, and this could be done using CPN.Fused Goal Functions (FCF) that contain encryption, QoS, and possibly energy, as illustrated below.SernCPN should be secure, but its QoS should be attractive to customers.
3. Energy utilization: when choosing particle trajectories, efficiency would be considered. A charge on the switches will be made to reduce power consumption, and traffic will be scattered on roads to reduce power consumption per packet or link [29].

The navigational provisions are reflected in an SDN-based SernCPN acquisition by determining the appropriate rules for the specified loads. An “oracle” would make the relevant decision, which would be powered with protection, QoS, energetic data, and stored in an Intellectual Security Memory (ISM). It would be built using Recurrent Neural Networks (RNNs) using a major learning technique such as Evolutionary algorithms in CPNs. A handler plugin would contain RNNs and ISM data used in machine learning. It should be noted that the RNN Reinforcement Learning (RL) based formative evaluation not only leverages current sensor readings.

Under the conditions described in II-B, ISM data will be classified into three parts. A great majority of user attributes, their definition for the evaluation process of destination paths and IP addresses, would be developed throughout the quality of the search.

It should concentrate on the statistical measures or assessments used by RNNs for recycling and then forward them to the SerIoT monitoring elements for monetization. Many methods or methodologies exist for traffic forecasting and detection systems [30]. Advanced threat segmentation methods would be selected at a later stage of the research. But it should begin with a range of topics that would give us a statistical perspective on the security connectivity of network devices.

(1) Assurance assessment (reliability testing) of systems connected to a SernIoT device: the probability of being the source or recipient of the attack. Reasonable precautions are considered in this category. Basic confirmations could involve the design of default passwords, and profiling firmware versions about the most modern security updates deployed, but more complicated.
(2) SFE Security (Trusted Level): a node is intended to be hacked, disabled, or monitored. It could take advantage of the level of accessibility of servers as a strategy to adjust the overall credibility of a circuit.
(3) Security for specific purposes is achieved through a variety of methods. The detection or use of assays, for example, is a basic indicator (lighter weight) that a given flow could be a component of assault.
  - Verify if the source or destination IP addresses of the stream are listed in a public list of forbidden IP addresses to be targets of aggression
  - Identifying flows over a predetermined limit
  - Access the IP address using the acquisition
  - Identify the plan for using nonstandard connections to apply effective techniques, such as comparing data traffic with attack methods using computational means or cognitive computation, at the end of the research
(4) QoS attributes are assigned through specific channels. The performance of specific lines, latency, instability, and loss levels should all be monitored by CPN.
(5) The energy consumption of certain nodes in terms of actual traffic volumes is determined.

Consequently, the FGF for SernCPN optimization would be created using the elements indicated in (1) to (5).

Intellectual Goal-Setting *G*(*f*, *P*) accepts nonnegative numerical actual values, where *f* symbolizes a flow (traffic defined by its source node, leading to impaired, broadcast address, and destination) and *P* represents a particular network route. Therefore, the quantities have been determined by the flow to which it relates the router cost. In either case, the decision system should search for a new flow path, leading to a lower *G* value. The entire function will also take QoS and effectiveness into consideration. It also describes responsibly the situations in which it is located.

The rejection level of flow *f* as in-circuit *e R* is now determined (*f*, *e*). The rejection factor is calculated using equations (1)–(3).

(1) $$\begin{array}{c} S\left(e,f\right)=\left\{\begin{array}{c} 0\;If\;RF\left(e,f\right)\leq SE\left(e,f\right)\\ Rejection\;Factor\left(e,f\right)-RE\left(e,f\right)\;Rejection\;Factor\left(e,f\right)>RE\left(e,f\right)\text{,} \end{array}\right. \end{array}$$

(2) $$\begin{array}{c} Rejection\;Factor\left(e,Q\right)=\underset{Q}{\sum }\begin{array}{c} S\left(e,f\right),or\;S\left(e,Q\right)=maximum\;of\;S\left(e,f\right)\\ \;Q \end{array}\text{,} \end{array}$$

(3) $$\begin{array}{c} H\left(e,Q\right)=Rejection\;factor\left(e,Q\right)+P\left(e,Q\right)+F\left(e,Q\right)\text{,} \end{array}$$

where *S*(*e*, *f*) represents circuit at reception level *e* of flow *f*; *Q* represents path; *P* and *F* are non-negative components.

**Figure 1:**
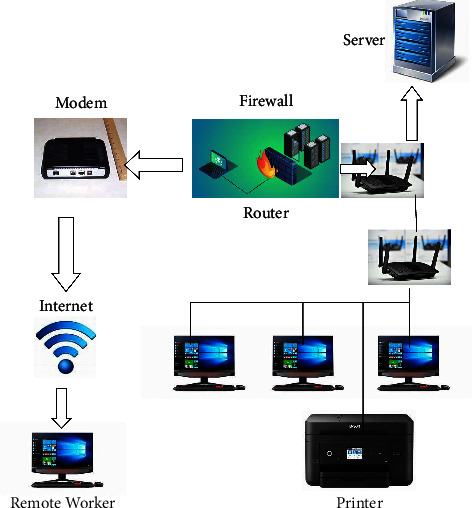
Sample diagram of basic single domain network.

Private Multihoming Protocol: as our reliance on digital grows, and the complexity of existing systems grows, it becomes increasingly difficult for a gadget to work alone. Cluster-driven interactions are required in this situation, which could be achieved via a range of approaches, including compound broadcast and multiplex. Each ensemble node must receive a point-to-point parcel when using compound multicast. Multipathing is a theoretically complex function that allows you to send a package to multiple devices while safeguarding your privacy by disseminating the message only as far as it has to go to impact each ensemble node, and only once on each path in Figure 1. To study shielded multiplex systems, numerous standards are developed, including ensemble affiliation maintenance; connect capacity absorption, receiver material demands, respondent workforce demands, and dependence on particular criteria.

**Figure 2:**
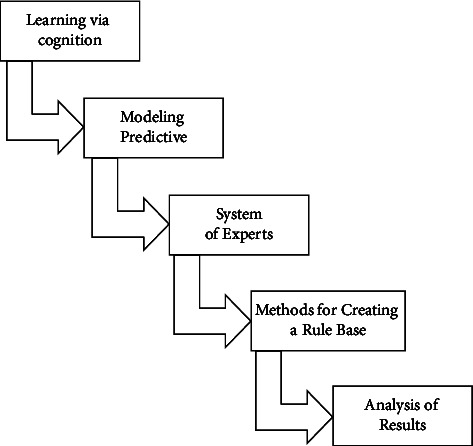
Steps to implementing context-aware computing.

If communications are required for an all-inclusive ensemble as well as for portions of a population, IIoT allowed safe multipath routing erupts. Protected multipathing is operationally better when the constellation would be sufficiently stationary due to the overhead (in Polynomial-based Key Management) of a larger number of rekeying routers every moment a node links or leaves that has a higher registration level than the lowest point as in cascade. They assume that multicast groups are always built using the core-based tree technique, in which each multicast pattern node has an unrevealed key that is shared with the central hub. It is worth noting that a central node is in charge of key distribution. A requirement for nodes to adopt multiple keys could express concern about node capabilities. Some nodes in an assorted ensemble have more attention resources than others. As a result, the requestor would first endorse a cluster controller before connecting to an ensemble to get any secrets. A validating router could act as a mediator, enabling new transactions to take place.  Table 1 highlights the elements of necessity and preliminary Figure 2.

## 4. System Architecture

Routing protocol techniques would be used to enhance a traditional SDN network. They would offer new SernCPN elements that are capable of performing activities required to achieve a research's objectives, especially intelligent safety-conscious forwarding. SernCPN's major parts are: SFE switches packages frequently by Open Flow regulations. SFE would also leverage the CPNs methodology to collect data on safety, quality of service, and energy demand.

SernCPN used a standard SDN controller in combination with SernCPN Navigation Engine (SRE). The RNN-based Intellectual Routing Module at the basis of SRE makes routing protocol using the methodology mentioned.

SernCPN combined SernCPN Routing Engine with a conventional SDN controller (ONOS–https://onosresearch.org/) (SRE). SRE's RNN-based Conceptual Routing Module creates congestion control using the methods presented.

SernIoT Honey trap is another platform that emulates the suitability of different products; it is linked to SernCPN and assesses the threats it receives. It could be taken by an adversary while causing damage to other SernCPN units.

In both IIoT setups or the communications system for transferring data to industrial integrated systems, a router is inserted into a circuit. It consists of a perturbation criterion, that would be used to train a system workplace to perform intrusion revelation on layer 3 (Internet layer, which could also endorse Multicast Broadband Service protocol or Network Technology) and layer 4 (information connection, that could endorse Multipath TCP) of a modus credo field, among data in a routing form of packets conveyed to web. It is worth mentioning that pathway description aggregates a network port's contents, which would be segregated into a series of packets containing the initial required information and customized to a chosen messaging protocol.

Unimaginable Particle Analysis enables network monitoring, controller provisioning, and security features, as well as constellation data gathering for outlier detection. A United States Patent and Trademark Act (UPA) makes it possible to determine who originated or received a data fingerprint containing specific sequences. Moreover, UPA could disclose node efficiency, incinerate network behavior, or support service providers in augmenting throughput or outcome. Packet processing rules and characteristics are provided in Tables 2 and 3.

Rigorous match: this type of resemblance involves exact comparability of detector element, i.e., a detector sector has one value provided in it.

Range match: an examination of a packet header to be in an array regulated by the classifier is known as a speculation match.

Protocol neutrality: a methodology that passes through a purifier should be independent, so that processing could be adjusted for different procedures or phases.

Protocol Fragmentation: a packet filter could handle packet decomposition. Effective regulation informs or audits: A design allows for integration or elimination of operations with the least amount of destruction to packet delivery. It keeps track of all entrance attempts, both hypothetical or blocked if required, as well as data that could be useful for study.

Considerations ranking: if a data set meets many syllabuses, packet criteria would allow objective relevance to be enforced on graded regulations, resulting in a distinct policy being in premise essential.

**Figure 3:**
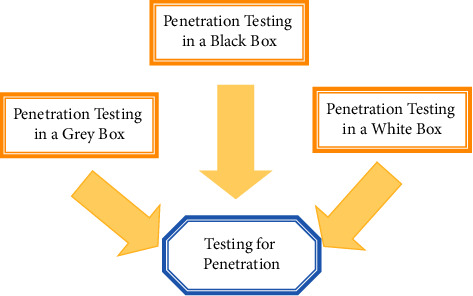
Analysis of vulnerability assessments.

SE process entails: (a) inspecting data to determine whether each of a predetermined set attributes of maliciousness exists; (b) determining a groove focused on the existence or lack of a set of rules in data, with merit/ratio delving deeper into the likelihood that data is abusive; or (c) dispensing to record based on data ratio. Support Vector Machine (SVM) is used in the suggested method, which would a popular technique for information categorization or advancement that has also been successfully used in malware detection. The SVM generates nonlinear restrictions in the flesh database by creating an evenhanded state border in euclidean space.  Figure 3 depicts a process flow diagram.

## 5. Implementation

SernCPN Routing Engine (SRE) would be redistributed as a plugin element in one or more SDN controllers, leveraging RNNs to build judgment oracles, allowing a semidistributed way of making judgments while leveraging the benefits of the SDN platform's semicentralization. A physical network topology would be replicated by attaching certain RNNs to SFEs. A single RNN's job would be to indicate, at the moment of decision, that the outlet unit should be used for a given SFE in the context of a flow with a particular destination. SRE would collect information in two aspects:

1. Clever Packets are an example of this (SPs)
2. through a supervisor that collects data on surveillance or diagnostic units

SerIoT, which would be based on the CPN concept, employs Intellectual Packets that go from one node to another or on their way to finding and collecting recorded network nodes. Typically, the SFEs of nodes accessed by CPs supply a CPs' path, while ACKs' path is origin rerouted from the destination address. The data generated by each CP that accessed the node could store or exploited by such hubs. A technique is amended in SernCPN since transmission of CPs or navigating throughout the network is managed by a regulator or SRE, so ACK messages, rather than flowing through the system using a path back to its source node, go to SRE that uses their information to determine navigation.

CPs would be used for information that would otherwise be unavailable, like connection delay or overall delay of neighboring nodes, including delay inside hubs, as well as data that could be provided directly by nodes (Web Sockets or demand) but is less essential (e.g., energy usage). CPs combine data to multiple sites along their journey and convey it to the supervisor in a short statement, decreasing communication costs.

Codesign would be the study of people interacting with workstations or to what extent workspaces or not urbanized to effectively communicate with humans and employees. One important element of codesign would be that different services with different outsets could replicate their cooperation and have different learning and customer attentiveness characteristics. Artificial intelligence, on the contrary, would be an imitation of general intelligence advancements using technology, primarily workstation PCs. Epistemology (gathering evidence or using syllabuses to expend data), sensation (using instructions to affect derivation or specific presumptions), or sympathetic activity are examples of these methods. Turing Test is a very well method for determining if technology could think like a person. They employed a classification algorithm to portray document matrices such that margins could be detected and used to tag different track assemblages. It is worth noting that while developing our approach, they took into account to General Data Protection Regulation (GDPR) published by European Union (GDPR). Several trials are performed to validate a proposed IIoT-enabled robust encryption.  Table 4 provides an overview of the experimental situation.

This appraisal is based on two sets of studies: (a) demonstration of recurring safety or (b) affirmation of resource-conscious safety.  Table 1 shows that possibility of detecting a hazard is dependent on a platform's ability to detect an abnormality appropriately. A criteria precision, detection ratio, or deceptive level of confidence could be used to evaluate methods. The condition of payload delivered across the boarding gate establishes corresponding statistics; this categorization is expressed by labels true/false positive/negative.

During the trial, they focused on gadget surveillance, configuration encounters, connectivity, infrastructure protection, and IIoT framework based on the test best habitat. They can isolate a probability of cyber threat forecast modeling using data collected for Table 4. They discover that gadget polymorphism might mimic the overall benefit of converting policies into maintaining levels, as distinct categories of assets might well be beneficial to combat associated dangers. Various methods/policies permit an indication of unique dissimilar peculiarities of the process to minimize false warnings depending on the exact quality requirements under monitoring or existing facts. Regularly enhance system conditions depending on efficiency challenges during the trial.

**Figure 4:**
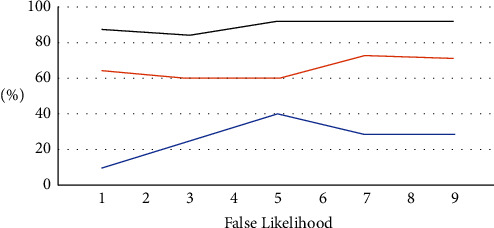
False and threats of likelihood.

**Figure 5:**
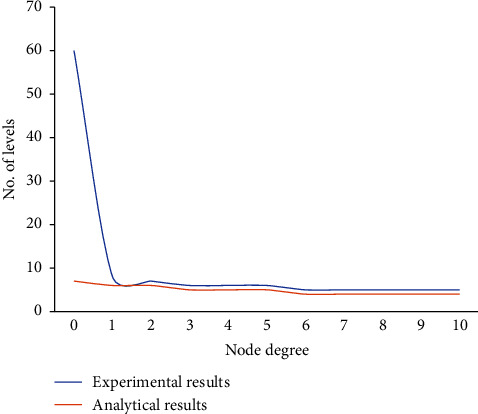
Results of experimental and analytical values for IIoT provision.

**Figure 6:**
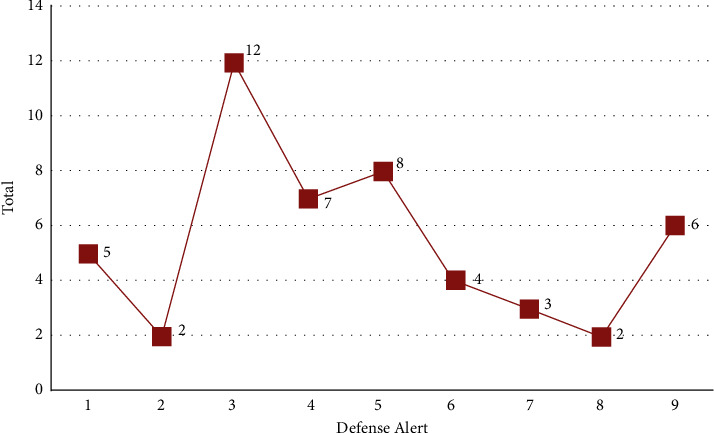
Alert processing time and success ratio.

For a myriad of purposes, a large number of different things attached to an IoT device creates significant security risks as shown in Figures 4–6. Imperfect experiences assess anticipated IIoT test-bed /debugging in terms of energy and information processing qualities. The chart shows an overview of simulator tests, that is developed to generate a generic threshold for security procedures in IIoT aligned anomalous mitigation context. The main goal of this experiment is to examine at edge nodes behaved during the outlier detection learning stage on the operating system, data tier, and Internet, as well as intrusion detection systems got better. A methodology assisted homeostatic nodes in handling a range of threats in a context-aware IIoT defensive scenario by providing an attempt to learn authority on allocating security capabilities within security components in Figure 4.

An overall handling period or security requirement fulfillment ratio is evidenced in Figure 5 based on the returning degree of security criteria for a variety of values of the system call's finishing time. It shows the results of scattering to defense mechanism on warning channels in this section. It constructed an instance because detected abnormalities are not always assaults, but could be a wide range of technical issues. It exploits by squeezing out alarm problems deliberately so that system components could be used for more relevant alerts. As a result, sorting is considered just as essential as abnormality detection. A suggested technique creates a stunning visual representation of the endangered service's security level. An accompanying chart illustrates the IIoT platform's security posture in an easy-to-understand manner. The handler could make predictions about hostile activities shown by the defensive warning axis in Figure 6. If an uneasy behavior is predictable, the context processor could check the alarms policy to ensure that event is rigorous (DAM classification).

**Figure 7:**
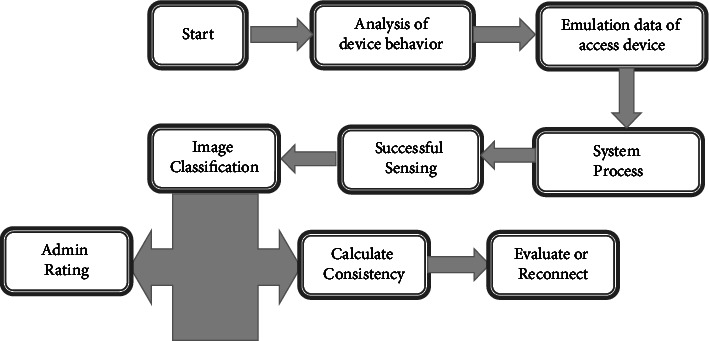
Modeling of notification trust.

The trust value notification modelled is shown in Figure 7. The time is taken to deliver data from the queue or construct a data frame not been estimated because it is extraneous to the data's range. To avoid connectivity or processing latency, set a 260 alerts/sec/zone criterion after a rigorous unit description study. This contract's receiving ratio of 9000 warnings per second is substantially higher than the gadget revealing that its technology can manage expandable IIoT networks with greater warning recurrence rates.

## 6. Conclusions

SernIoT is to enhance a wide range of security techniques targeted to a quickly evolving IoT ecosystem. A key emphasis of techniques given in the study is attributed to the SernIoT initiative by authors is on novel protocol methods in terms of quality and integrity. The impact of route discovery on the security of IoT devices linked to signal integrity core network equipment is evaluated. These features of network activity via IoT devices would allow us to more clearly characterize dangers and indications of malicious actions than in a general network. The proposed model is designed for security-related data across multiple risks and possible attacks. As a result, the RNN component could be used as an intelligent tool for making appropriate decisions in a dynamic environment. Protection represents an edge node and the analysis predicted remedies for mitigating security risks. The proposed system enhanced security capabilities or programs with secure or user-friendly graphical user interfaces. A potential mitigation mechanism reduces the likelihood of safety threats and incidents. In a moderate approach, effective self-governing or parasympathetic protection displayed a positive influence on overall system efficiency. The focus of this study is on identifying potential risks, impacts, or dangers, and preventive actions for IIoT systems. The goal of our investigation purpose is to provide a framework to understand or monitor security vulnerabilities in the IIoTdefense shield.

## Figures and Tables

**Table 1:** Parameters of necessity.

Title	Unit	Scheme assumed
Operational simplicity	Set the timer interval	Clusters of compound compounds
Intensity communications in real-time	Buffer for addresses	Enable
Depletion of resources	Distributing access	There are several entries for each link.
Per message connection rate	A network that is only loosely connected	Extreme

**Table 2:** Environmental parameters filtering.

Parameters	Units
Size per packets	36 bits
Route time	2000 ms
Simulated kit	ESP8266
Purpose	12
Latency	120 ns
Input	300 megabytes per second
Packet filter for nonlinear	50 megahertz
Destination port	500

**Table 3:** Criteria for evaluating malware.

Malware scoring principles		Malware grouping rankings	
Average	Storyline	Parameters	Description
Launch	The program component is on the verge of being implemented.	Serious	Storage is disrupted.
Circulation	The cipher has been disseminated.	Hazardous	Bandwidth in distress
Level of resentment	The payload is connected to the set on.	Negligible	Easily accessible and useful

**Table 4:** Environment for trial and error.

Parameters	Units
Test	Regular grid
Configurations	5
IIoT sensors	150
Node capacity	50 GB
Storage capacity	20 TB
Pins	20
SoC	Adafruit FONA
Network	802.11 bgn
Antenna model	Low power omni direction
Signal propagation	Antenna with spring
Software	Arduino IDE
Cloud	Microsoft
RAM	DDR–3, 4 GB
Processor	Quad-core
Port number	8
Payload	60 bits
Duration	150 mins
Number of events	6000 per 15 minutes
Humidity	25%
Temperature	−3 minute

## Data Availability

The data shall be made available from the corresponding author upon request.
